# Proteomic analysis identifies HMGA2 as a novel biomarker of overall survival in papillary renal cell carcinoma

**DOI:** 10.1002/cam4.6077

**Published:** 2023-06-07

**Authors:** Tian Wei, Huiya Xu, Huishan Liang, Yating Lian, Huiyu Chen, Zhangyi Zheng, Minghui Zhong, Junfeng Liu, Ran Wang, Fen Wang

**Affiliations:** ^1^ Department of Pathology The First Affiliated Hospital, Sun Yat‐Sen University Guangzhou PR China; ^2^ Department of Pathology, Sun Yat‐Sen Memorial Hospital Sun Yat‐Sen University Guangzhou PR China; ^3^ Department of Pathology The Seventh Affiliated Hospital, Sun Yat‐Sen University Shenzhen PR China

**Keywords:** HMGA2, papillary renal cell carcinoma, prognosis, proteomics, TCGA

## Abstract

**Purpose:**

Although papillary renal cell carcinoma (PRCC) has a relatively favorable prognosis, a small number of patients with lymph node or distant metastasis have a poor prognosis. Owing to the complex typing and heterogeneity of PRCC, it remains difficult to provide risk stratification. The objective of our research was to identify potential markers of PRCC prognosis.

**Methods:**

We performed proteomics and bioinformatics analyses on six pairs of formalin‐fixed paraffin‐embedded tumor and paired normal tissue samples. The Cancer Genome Atlas (TCGA) data were used to analyze the prognostic value of differentially expressed proteins (DEPs) in PRCC. We verified the expression of the major biomarker through immunohistochemistry (IHC) in 91 PRCC tumor specimens.

**Results:**

Proteomic analysis revealed 1544 DEPs between tumor and paired normal tissues. PRCC transcriptomic data from the TCGA database revealed that compared to non‐tumor tissues, the expression of high‐mobility group protein A2 (HMGA2) was upregulated in tumor tissues, and patients with high HMGA2 expression exhibited shorter overall survival times. HMGA2 was associated with PRCC tissue subtype and higher cell pleomorphism. Both TCGA and IHC results showed that HMGA2 expression was associated with lymph node metastasis and clinical stage.

**Conclusion:**

HMGA2 was positively correlated with malignant progression and could be a valuable novel prognostic biomarker for PRCC risk stratification.

## Graphical abstract

Through proteomics and bioinformatics analyses of PRCC, we found that high‐mobility group protein A2 (HMGA2) was highly expressed in tumor tissues, and patients with high HMGA2 expression exhibited shorter survival times. In addition, HMGA2 expression was associated with tissue subtype, lymph node metastasis, and clinical stage. This study demonstrated for the first time the potential relevance of HMGA2 as a prognostic biomarker in PRCC, which could improve patient stratification and inform its clinical management.

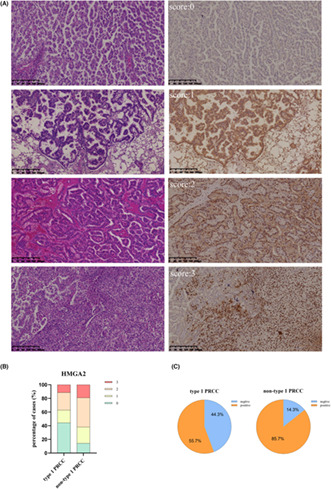

## 1 INTRODUCTION

Papillary renal cell carcinoma (PRCC), the second most common subtype after clear cell RCC (ccRCC), accounts for approximately 13%–20% of RCC cases.^1^, ^2^, ^3^ Previous studies subclassified PRCC into Types 1 and 2, but it is now believed that Type 2 PRCC may include tumor entities such as MiT family translocation RCC and FH‐deficient RCC.^4^ According to molecular stratification, PRCC can be divided into mesenchymal–epithelial transition factor gene *MET*‐driven or *MET*‐independent subgroups, which provided a basis for effective targeted therapies, such as multiple tyrosine kinase receptors inhibitor cabozantinib.^5^ New therapeutic strategies have improved the 1‐year‐survival rates of PRCC patients by ~75%.^6^ However, the prognosis is still poor for the small proportion of patients.^7^, ^8^ The 2023 Genitourinary Cancers Symposium (ASCO GU 2023) has shown that immunotherapy and cabozantinib were effective and safe in the treatment of advanced RCC, but these study are still ongoing. Due to the complex typing and heterogeneity of PRCC, it remains difficult to provide risk stratification and identify relevant prognostic markers, which is highly desirable for effective disease management.

Proteomics‐combining mass spectrometry (MS)‐based and bioinformatics approaches has emerged as a powerful platform for identifying useful biomarkers.^9^, ^10^ Compared with genomics and transcriptomics, proteomics is used to directly comprehensively analyzed determine protein abundance and elucidate protein‐specific regulatory mechanisms. This “post‐genome‐era” technology has been of great importance not only in elucidating tumor behavior and pathogenesis, but also in the development of diagnostic, prognostic, and targeted treatment strategies. For example, a proteomic map of diffuse‐type gastric cancer has provided a comprehensive view of the altered proteome and related signaling pathways.^11^ In the analysis of Chinese patients with ccRCC, a proteogenomic approach was used to identify a biomarker for poor prognosis and revealed that metabolic disorders and an exaggerated immune response were associated with high mortality rates.^12^ Xu et al. identified potential drug targets and prognostic biomarkers by utilizing a proteomics analysis of tissues from 103 Chinese patients with lung adenocarcinoma.^13^

Bioinformatics analysis using public databases can further be applied to link proteome markers with clinical data and ultimately generate novel strategies for disease management. Tumor bioinformatics resources provide access to multicenter genome, transcriptome, and proteome data that can be used to determine molecular‐level differences between cancers. Using three acute myeloid leukemia (AML) RNA‐sequencing (RNA‐seq) cohorts (BeatAML, LeuceGene, and The Cancer Genome Atlas [TCGA]), Lee et al. identified a novel biomarker for AML treatment that targets apoptosis and cancer pathways.^14^ In a study of 782 metastatic melanoma specimens, Hahn et al. found that the mutations of DNA and the variations of copy number were not associated with body mass index across TCGA cohorts.^15^ Using RNA‐seq data from 530 patients with ccRCC in TCGA, Li et al. identified a novel micropeptide associated with diagnosis and treatment.^16^ Bioinformatics resources have contributed substantially to basic tumor research and clinical treatment.

In this article, the proteomic landscape of PRCC were analyzed by proteomic analysis applied in tumors and their corresponding non‐tumor specimens in six PRCC patients. Using a bioinformatics approach combined with TCGA database, we screened the differentially expressed protein (DEP) related to prognosis of PRCC. To verify the expression of the DEP in different subtypes of PRCC, we performed immunohistochemistry (IHC) on 91 PRCC tissues. To sum up, our data may identify a potential marker for risk stratification in PRCC.

## 2 MATERIALS AND METHODS

### 2.1 Patient samples

Our study protocol was approved by the Institutional Review Board of The First Hospital of Sun Yat‐sen University, Guangzhou, China (No. [2021]404). IHC was performed on 91 formalin‐fixed and paraffin‐embedded tumor tissues and paired adjacent normal tissues of PRCC obtained from the Pathology Department of the First Affiliated Hospital, Sun Yat‐sen University. All patients were diagnosed and received surgery between January 2012 and December 2019. We numbered all samples and randomly selected six cases for proteomics analysis of cancer and paired adjacent normal tissues (~0.5 cm from the edge of the tumor) in paraffin‐embedded tissues.

### 2.2 TMT proteomics and bioinformatics analyses

Tandem mass tag (TMT) proteomics analysis was conducted as previously described.^17^ TMT proteomics analysis involves protein extraction, trypsin digestion, TMT labeling, high‐performance liquid chromatography fractionation, liquid chromatography–tandem mass spectrometry (LC–MS/MS), and a database search. For each group, principal component analysis and unsupervised hierarchical clustering were used to remove outliers. Bioinformatics was applied to evaluate the functional annotation and enrichment of the DEPs using online databases. First, Wolfpsort software (version 0.2) was utilized to predict the subcellular localization of the screened proteins. We converted the screened protein IDs into UniProt IDs to perform Gene Ontology (GO) annotation of the DEPs based on three categories: biological process, cellular component, and molecular function. The GO annotation proteome was obtained from the UniProt‐GOA database (http://www.ebi.ac.uk/GOA/). We used the Kyoto Encyclopedia of Genes and Genomes (KEGG) database to annotate and match the screened proteins to their associated signaling pathways using the online tool KAAS (version 2.0).

### 2.3 TCGA data processing

We downloaded the data of transcriptome profiles and the clinical information of the PRCC cohort from TCGA data portal (https://tcga‐data.nci.nih.gov/tcga/), and total of 289 PRCC patients' data was finally used in this study. The RNA sequence profiles were stored in transcripts per million (TPM) tracking format and log_2_‐transformed. Differentially expressed genes (DEGs) between cancer and adjacent tissues were obtained by using DESeq2 R package. The transcriptome and translatome expression levers were analyzed using Pearson's correlation. The expression of SOSTDC1, HMGA2, and FHL1 was analyzed in PRCC tumor samples and their corresponding normal tissues of 31 patients using the TCGA dataset. The R software (version 3.6.3) was used to perform the data of visualizations.

### 2.4 Immunohistochemistry

We retrieved deparaffinized antigens under high pressure and temperature in sodium citrate buffer (pH 6.0) for 3 min. The slides were incubated with anti‐HMGA2 (1:400; CST) overnight at 4°C, and then washed in PBS buffer for three times, and incubated with IHC secondary antibody (an anti‐mouse/rabbit Kit form Gene Tech) following the product instructions. A digital pathology slide scanner (KFBIO) was used to analyze the intensity of the staining according to the following grading: 0 as negative, 1 as weak, 2 as moderate, and 3 as strong, with positive areas defined as 1 (<10%), 2 (10%–49%), 3 (50%–75%), and 4 (>75%). An immunohistochemical composite staining index was calculated for the whole tissue sections by multiplying the intensity and positive area scores, which generated values between 0 and 12. The final staining index of 0–1 was scored as 0, 2–4 as 1+, 5–7 as 2+, and 8–12 as 3+. For example, a case that scored 3 for area and 2 for staining intensity had a final score of 2+. Immunohistochemical staining was scored as either 0–1, representing low expression of HMGA2, or 2–3, representing high expression. Attention should be paid to the differentiation of the hemosiderin staining (Figure S2B). Human colon cancer tissue was selected as a positive control, in accordance with the instructions provided with the antibody product. The negative control were the adjacent normal tissues, and the pictures of adjacent normal tissues are showed in Figure S2C.

### 2.5 Statistical analysis

We used SPSS software (version 25.0; IBM SPSS) to perform statistical analyses, and the clinical information was analyzed by standard statistical tests, including Student's *t*‐test and the chi‐square test, among others. We tested the association between survival rates and the DEGs using a Kaplan–Meier model, with *p*‐values and HRs computed by log‐rank and Cox regression analyses. Univariate Cox regression analyses were used to filter potential prognostic indicators. Significance was set at *p* < 0.05.

## 3 RESULTS

### 3.1 Quantitative analysis of proteins by tandem mass tag‐labeled liquid chromatography–tandem mass spectrometry

**FIGURE 1:**
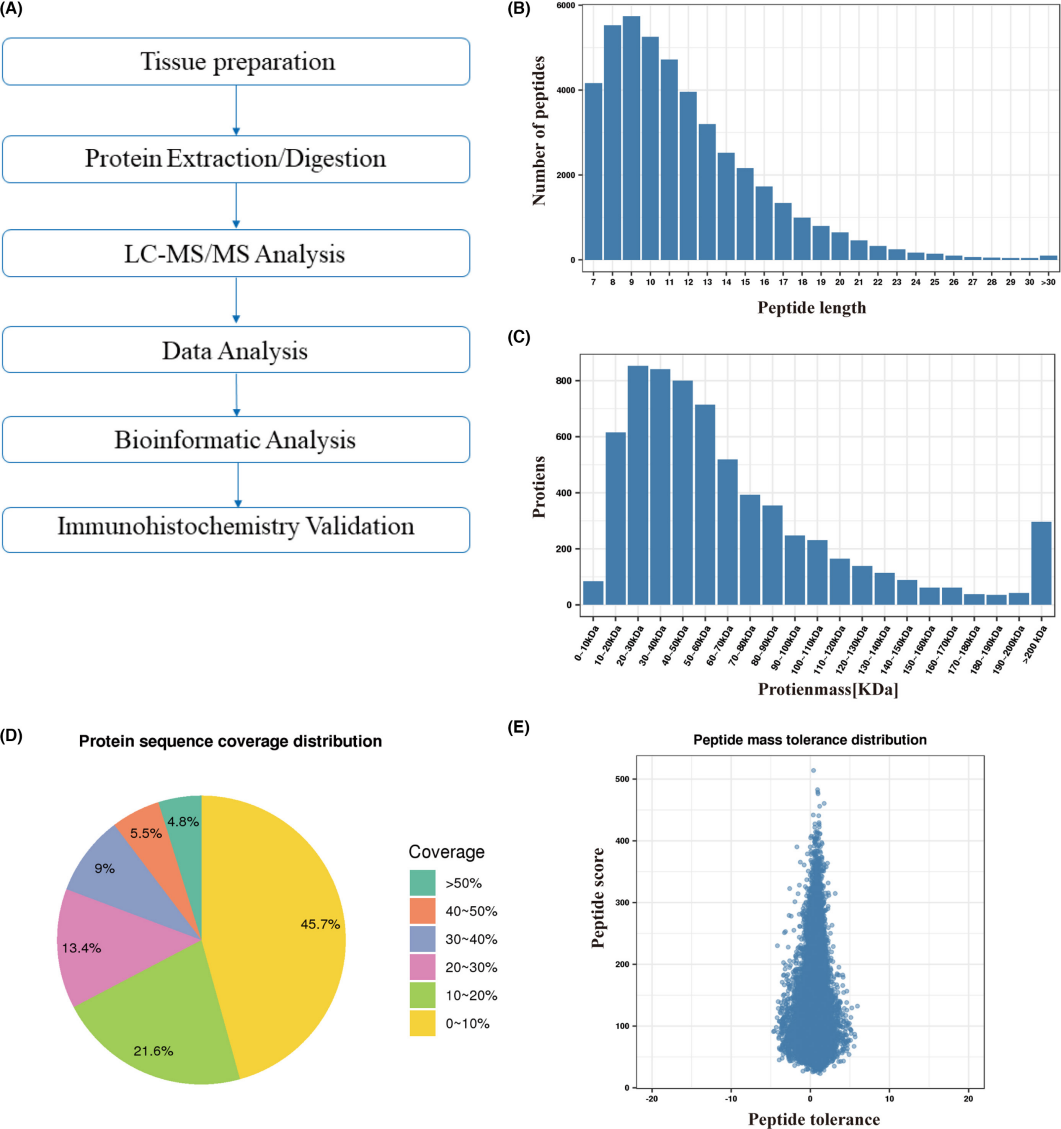
Overview of the proteomics analysis of papillary renal cell carcinoma. (A) General workflow for the liquid chromatography–tandem mass spectrometry (LC–MS/MS) analysis coupled with tandem‐mass tag reagents labeling. (B) Length distribution of the identified peptides. (C) Molecular weight distribution of identified proteins. (D) Protein coverage distribution of the identified proteins. Based on shotgun (also called bottom‐up) strategy, MS preferentially scans peptides with higher abundance. Coverage is the ratio of the sum of the lengths of all unique peptides identified for a protein to the total length of the protein. (E) Mass error (−10–0 ppm) distribution of identified peptides. The reliability of peptide identification was negatively correlated with the distribution of quality deviation.

We determined the protein profiles in PRCC by TMT‐labeled LC–MS/MS of matched tumor and normal tissues obtained from six specimens of surgically resected formalin‐fixed paraffin‐embedded PRCC tumors (Figure 1A). Among the six patients aged 34–77 years (the median age was 55 years), four were male and two were female. These six cases were Type 1 PRCC with similar histomorphology. A total of 268,810 spectra were obtained, corresponding to 5285 proteins (Table S1). Most peptides ranged from 7 to 20 amino acids in length, indicating that the proteins were completely digested during sample preparation (Figure 1B). Because of their poor solubility, higher‐molecular‐weight proteins (>100 kDa) were retained during preparation, thereby providing abundant information for macromolecular protein analysis (Figure 1C). In the eucaryote whole proteome, most proteins have a low abundance and low coverage rate. Our results showed that most proteins had coverage rates ranging from 1% to 20%, with 34.1% of the 5285 proteins having a coverage exceeding 20% (Figure 1D). Among the identified peptides, most of mass errors were distributed range from −10 to 0 ppm (Figure 1E). As expected, most spectra's first‐order mass error was within 10 ppm that accord with the high precision characteristics of MS.

### 3.2 An analysis of differentially expressed proteins between PRCC tumors and paired normal tissues by bioinformatics

**FIGURE 2:**
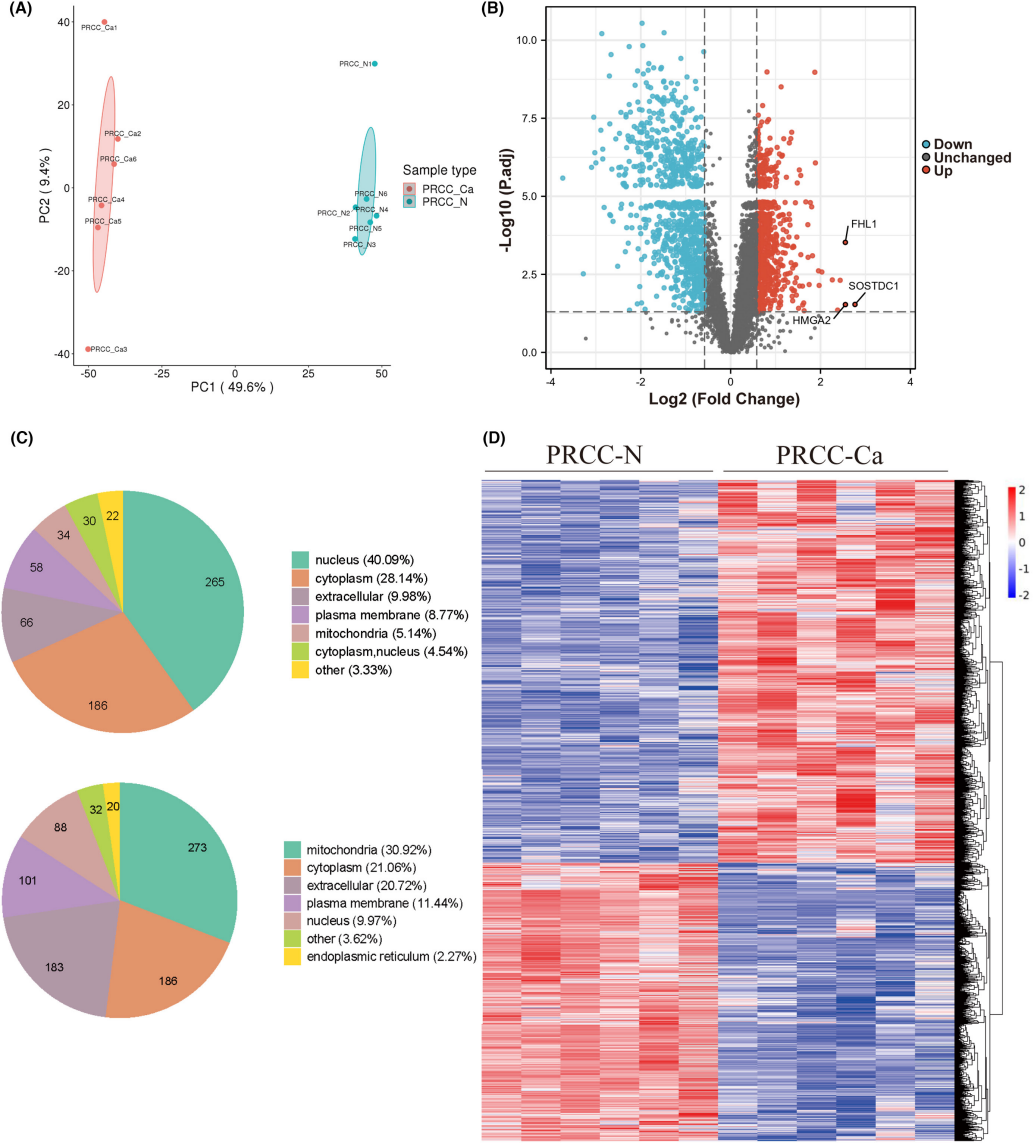
Hierarchical cluster analysis of differentially expressed proteins (DEPs) in paired papillary renal cell carcinoma with their corresponding adjacent normal tissues. (A) Principal component analysis illustrating the moderate clustering of samples within two subtypes. The better the degree of aggregation between repeated samples, the better the quantitative repeatability. (B) Volcano plot highlighting the DEPs in PRCC‐Ca versus PRCC‐N. (C) Subcellular location of the DEPs in PRCC‐Ca versus PRCC‐N. Some major constituents of eukaryotic cells are: extracellular space, cytoplasm, nucleus, mitochondria, endoplasmic reticulum, and plasma membrane. (D) Heat map of all DEPs identified between PRCC‐Ca versus PRCC‐N. The tree diagram on the right side represents the results of cluster analysis of different samples from classification.

This analysis was performed using hierarchical clustering, principal component analysis, and Pearson's correlation coefficient; it showed a good repeatability between PRCC cancer tissues (PRCC‐Ca) and paired adjacent normal tissues (PRCC‐N) (Figures 2A and S1). With *p*_*adj*_ at 0.05 and fold‐change of 1.5, we detected 1544 DEPs, including 661 upregulated and 883 downregulated proteins (12.50% and 16.70% of 5285, respectively) (Figure 2B and Table S2). The majority of the upregulated DEPs were located in the nucleus (265 proteins, 40.09%), cytoplasm (186 proteins, 28.14%), extracellular space (66 proteins, 9.98%), and mitochondria (34 proteins, 5.14%), all of which are essential functional components of cells (Figure 2C). A heatmap was generated to present the DEPs between PRCC‐Ca and PRCC‐N (Figure 2D). We selected the top three DEPs (SOSTDC1, HMGA2, and FHL1) with the highest PRCC‐Ca versus PRCC‐N expression ratio as candidate biomarkers, which were represented in 83.33% (5/6) of our samples.

### 3.3 Validation of MS data by TCGA data

**FIGURE 3:**
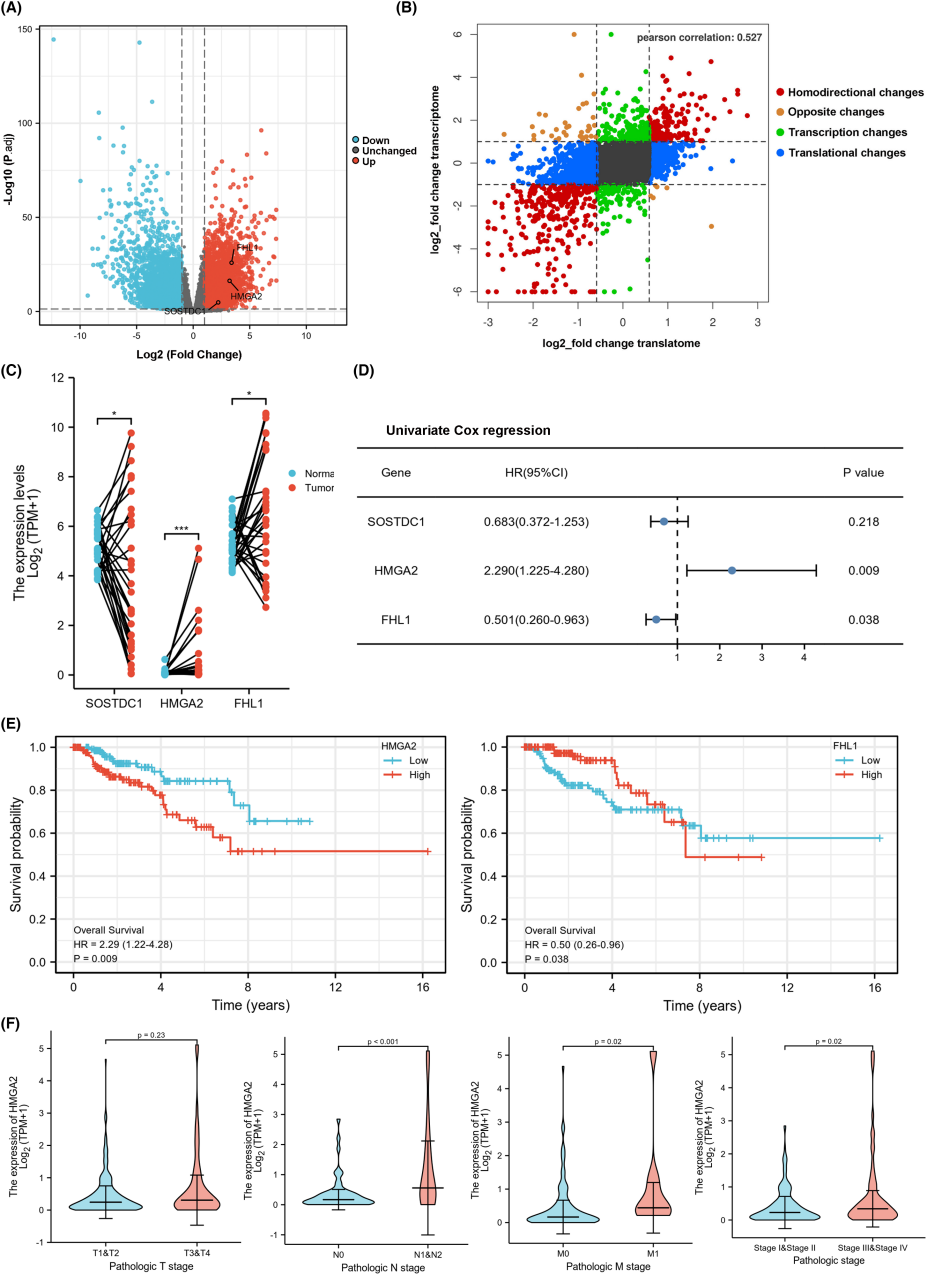
Validating candidate biomarkers in transcriptomic profiles for PRCC via TCGA datasets. (A) Volcano plot presented up‐regulated and down‐regulated DEPs (fold change ≥2; *p* < 0.05) in 289 PRCC patients from TCGA database. The top 3 DEPs (SOSTDC1, HMGA2, and FHL1) were marked out. (B) Scatter plot of 9‐quadrant analyses showed the correspondence between transcriptome and proteome in PRCC patients. (C) The expression of SOSTDC1, HMGA2, and FHL1 in tumor and paired‐normal samples from 31 PRCC patients in TCGA database. (D) Univariate Cox regression analyses were visualized in the forest plots to show the valuable prognostic biomarker of a set of individual genes between SOSTDC1, HMGA2, and FHL1 for PRCC. SOSTDC1 wasn't the independent prognostic factor. (E) *p*‐values and HRs were computed by log‐ rank test and Cox regression to draw Kaplan–Meier (KM) curves which showed the overall survival analysis of HMGA2, and FHL1. (F) Correlation analysis of HMGA2 expression and clinicopathological features which obtained from the TCGA database.

In order to examine the concordance between the expression levels of transcripts and proteins, we analyzed the RNA‐seq profiles of 289 PRCC patients from the TCGA database. The amount of DEGs totaled 17,790 were screened with a fold change >2 and adjusted *p* value <0.05, of which the upregulated genes were 6693 and the downregulated genes were 4404 (Figure 3A and Table S3). We used Pearson's correlation to assess the relationship between the transcriptome and translatome expression levels in PRCC. A nine‐quadrant associate analysis revealed that proteomic changes were positively correlated with transcriptional/gene changes in PRCC (*r* = 0.527; Figure 3B). We found that the gene expression of SOSTDC1, HMGA2, and FHL1 was significantly higher in tumor tissues than their paired adjacent normal tissues from 31 PRCC patients in TCGA database (Figure 3C), especially that of HMGA2 (*p* < 0.001).

Next, we verified the prognostic value of these three candidate biomarkers. Univariate Cox regression analysis revealed that HMGA2 and FHL1 were independent prognostic factors for PRCC, and that HMGA2 may be associated with poor survival (HR = 2.290, *p* < 0.05; Figure 3D and Table S4). According to the overall survival analysis, patients with high levels of HMGA2 expression had shorter survival times (Figure 3E). Furthermore, HMGA2 expression was linked to lymph node metastases (*p* < 0.001) as well as distant metastases (*p* = 0.02), and pathologic stage (*p* = 0.02) (Figure 3F). Therefore, PRCC transcriptomic data from TCGA database identified HMGA2 as a potential prognostic marker in PRCC.

### 3.4 Immunohistochemical analysis of 91 PRCC tissue sections

Immunohistochemistry was used to examine HMGA2 protein expression in 91 surgically resected PRCC specimens. The clinicopathological information for the 91 patients is shown in Table 1. Among patients in the Type 1 cohort, the median age was 52 years (range: 22–82 years), and 55.5 years (range: 32–79 years) in the non‐Type 1 cohort. We did not observe differences in protein expression between the cohorts or sexes. The incidence of tumor Stages 1 and 2 varied between the Type 1 group (64 [91.4%] and 6 cases [8.6%], respectively) and the non‐Type 1 PRCC group (14 [66.7%] and 6 cases [33.3%], respectively; *p* = 0.004). We identified six cases with lymph node metastases, five of which were non‐Type 1 PRCC, also showing statistical significance.

**TABLE 1:** Clinicopathological features of the PRCC cases.

	Type 1 of PRCC (*n* = 70)	Non‐Type 1 of PRCC (*n* = 21)	*p*‐value
Age^a^	52.0 (22–82)	55.5 (32–79)	0.152
Gender			
Female	8 (11.4%)	4 (19.0%)	0.365
Male	62 (88.6%)	17 (80.0%)	
Nephrectomy			
Partial	53 (75.7%)	5 (23.8%)	<0.001
Total	17 (24.3%)	16 (76.2%)	
Tumor size (cm)^a^	4.2 (0.2–11.5)	6.0 (2.0–15.0)	0.004
T stage (at nephrectomy)			
pT1	64 (91.4%)	14 (66.7%)	0.004
pT2	6 (8.6%)	7 (33.3%)	
pT3	0	0	
Regional lymph nodes (at nephrectomy)			
N0	69 (98.6%)	16 (76.2%)	<0.001
N1	1 (1.4%)	5 (23.8%)	
Distant metastasis (during follow‐up)			
M0	70 (100%)	21 (100%)	–
M1	0	0	
Recurrence			
Positive	0	0	–
Negative	70(100%)	21(100%)	

*Note*: –, no statistics were computed.

^a^ Median (range).

**FIGURE 4:**
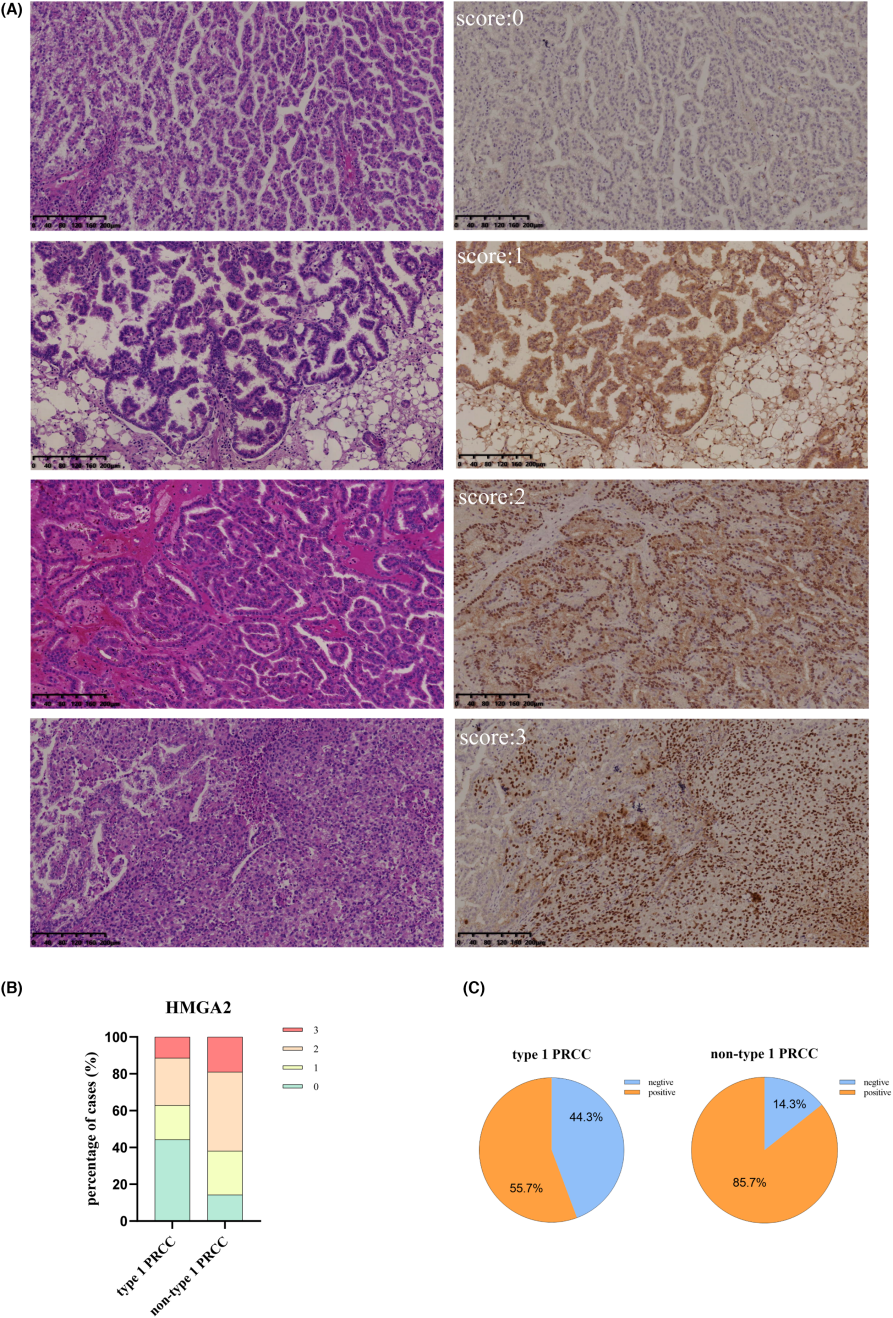
Typical immunohistochemical staining samples and the corresponding staining scores of HMGA2 in PRCC tissues. (A) Immunohistochemical and corresponding hematoxylin and eosin (H/E) staining on PRCC separately with Score 0, 1, 2, 3. HMGA2 expression was localized in the nucleus (scale bars: 200 μm). (B) Distribution of the staining scores of HMGA2 in type1 PRCC and non‐type 1 PRCC. (C) Positive rate of HMGA2 between Type1 PRCC and non‐Type 1 PRCC.

Immunoreactivity for HMGA2 is located in nuclei and negative in adjacent normal tissues. The original staining scores of whole tissue sections of PRCC are shown in Figure S2A. Representative examples of HMGA2 staining are shown in Figure 4A. In total, 66.7% (2/3) of the 91 cases of PRCC tumor were positive. Based on the PRCC composite scores, we observed higher rates of HMGA2 positive staining in non‐Type 1 (85.7%) than Type 1 tissues (55.7%, *p* < 0.05; Figure 4B,C). Most Type 1 PRCC cases presented weak‐to‐moderate staining of the nuclei, but nuclei staining intensity was usually strong in non‐Type 1 PRCC tissues. With hematoxylin and eosin staining, we found that strongly stained tissues had greater cellular atypia, as evidenced by larger nuclei and irregular nuclear membranes. Collectively, based on our findings, HMGA2 expression appears to be correlated with PRCC tissue subtypes, cell pleomorphisms, and significantly associated with clinical stage and lymph node metastasis (Table 2).

**TABLE 2:** Correlation between HMGA2 expression and the clinicopathological characteristics of the PRCC patients.

Characteristics	Number of cases (%)	Low expression no. (%)	High expression no. (%)	*p*‐value
Gender				
Female	12 (13.2)	7 (58.3)	5 (41.7)	0.929
Male	79 (86.8)	45 (57.0)	34 (43.0)	
T stage				
pT1	78 (85.7)	44 (56.4)	34 (43.6)	0.729
pT2	13 (14.3)	8 (61.5)	5 (38.5)	
pT3	0	0	0	
Regional lymph nodes				
N0	85 (93.4)	52 (61.2)	33 (38.8)	0.005
N1	6 (6.6)	0	6 (100)	
Distant metastasis				
M0	91 (100)	52 (57.1)	39 (42.9)	–
M1	0	0	0	
Recurrence				
Positive	0	0	0	–
Negative	91 (100)	52 (57.1)	39 (42.9)	
Clinical stage				
I–II	85 (93.4)	52 (61.2)	33 (38.8)	0.005
III–IV	6 (6.6)	0	6 (100)	
Type				
Type 1	70 (76.9)	44 (62.9)	26 (37.1)	0.044
Non‐Type 1	21 (23.1)	8 (38.1)	13 (61.9)	

*Note*: –, no statistics are computed.

### 3.5 Analysis of DEPs' functional enrichment

**FIGURE 5:**
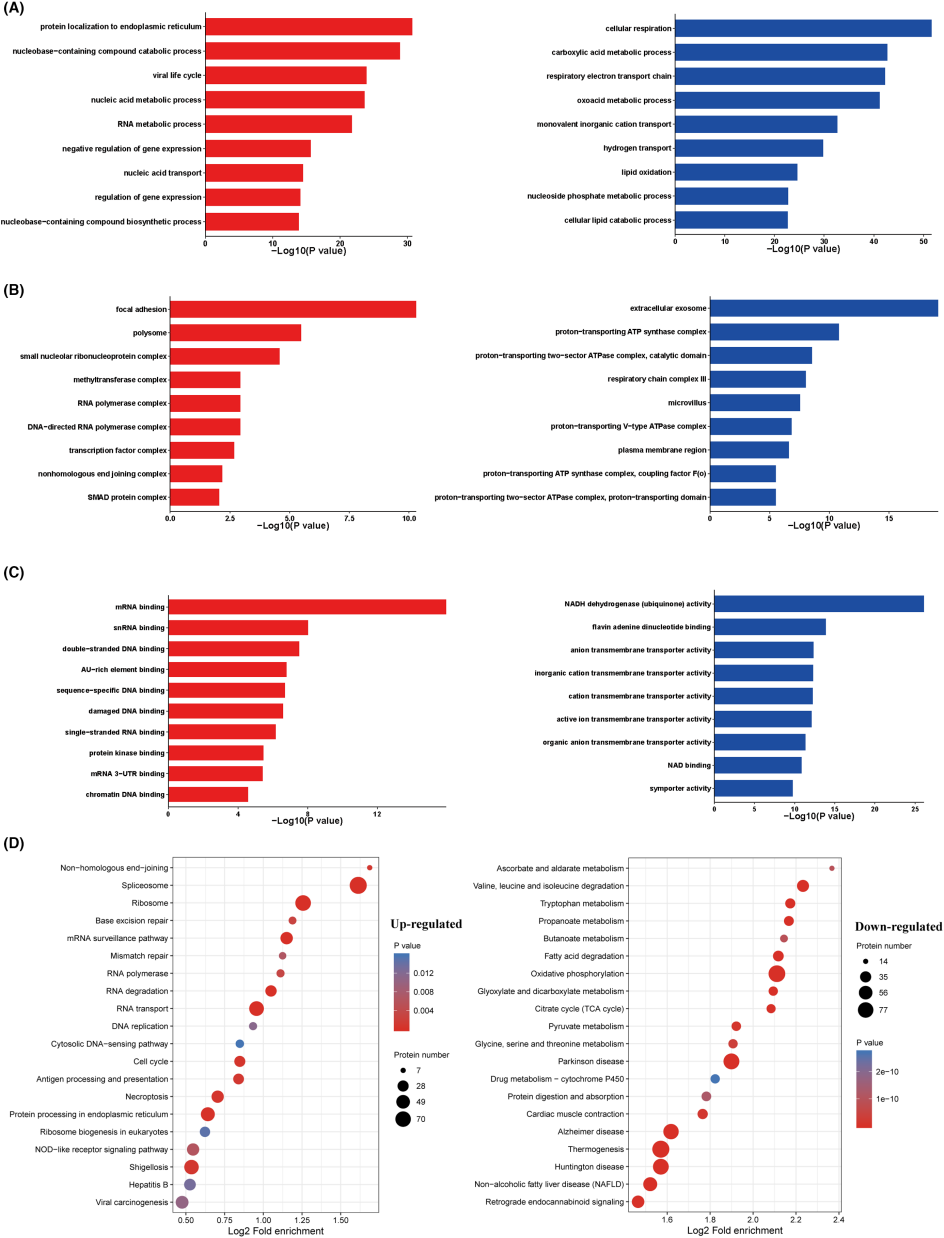
Gene Ontology (GO) annotation and KEGG pathway of differentially expressed proteins (DEPs). The DEPs between PRCC‐Ca versus PRCC‐N were classified by GO annotation contained biological process (A), cellular component (B) and molecular function (C) (red: upregulated; blue: downregulated). (D) Major enriched KEGG pathways of DEPs (left: upregulated KEGG pathways; right: downregulated KEGG pathways). A corrected *p*‐value <0.05 is considered significant.

We performed functional enrichment analyses of the DEPs using the GO and KEGG databases (Tables S5 and S6). According to Fisher's exact test, we identified functional categories and pathways that showed a significant enrichment (*p* < 0.05). Biological process annotation indicated that the negative regulation of gene expression and metabolic processes of nucleic acids, RNA, DNA, and nucleobase‐containing compounds were significantly enriched in the tumor proteome (Figure 5A–C). In contrast, cellular respiration, cellular macromolecule biosynthesis, and respiratory electron transport chains (Figure 5A) were enriched in the matched normal tissue proteomes. HMGA2 was involved in the regulation of gene expression and metabolic processes of nucleic acids, RNAs, and DNAs. According to KEGG analysis, the DEPs played key roles in spliceosome, ribosome, and RNA transport (Figure 5D), all of which are involved in gene transcription and translation.

## 4 DISCUSSION

By screening DEPs using proteomics technology and TCGA data, we investigated biomarkers associated with poor prognosis in PRCC. Immunohistochemistry of 91 pairs of PRCC tissues demonstrated that HMGA2 was associated with PRCC tissue subtype and was positively correlated with clinical stage and lymph node metastasis. The results of this study provide that HMGA2 may be a prognostic biomarker for PRCC for the first time.

Before the World Health Organization's (WHO) 2022 tumor classification was published, PRCC was categorized into Types 1 and 2. Type 1 PRCC consists of papillae and tubular structures surrounded by small cells with small oval nuclei and basophilic cytoplasm, Type 2 PRCC, on the other hand, is more heterogeneous and features papillae covered with large, eosinophilic cells and spherical nuclei with prominent nucleoli. The outcome of patients with PRCC Type 2 is worse than that of patients with PRCC Type 1.^18^ Since many papillary carcinomas cannot be classified under the existing criteria, this classification is unsatisfactory. As molecular evidence continues to accumulate, Type 2 PRCC appears genetically heterogeneous may be further subdivided based on its genetic and molecular makeup, clinical characteristics, and prognoses.^19^ Therefore, based on the relatively consistent morphology and genetics of Type 1 PRCC and the new WHO classification system, we divided PRCC into Type 1 and non‐Type 1 for our analysis. Compared with Type 1 PRCC, the levels of HMGA2 were higher in non‐Type 1 PRCC. We also found that HMGA2 expression may be positively correlated to the malignant progression of PRCC, which corresponds with the poorer prognosis observed for non‐Type 1 compared with Type 1 PRCC.

Optimizing therapeutic protocols would be easier if prognosis could be accurately predicted. The prognosis of PRCC is closely associated with its clinical stage and histopathological features. Histological, staging, and clinical variables were shown to be prognostically relevant in the 2012 WHO/ISUP grading system.^20^ A recent study examined the trends in prognosis of 87 PRCC cases. A poor prognosis was associated with a larger mass, lymph nodes metastasizing, distant metastasizing, a higher stage, and higher pathological grade.^21^ As the molecular characterization of PRCC has progressed over the past few years, including its oncogenes, microRNAs, and long non‐coding RNAs, though no reliable molecular biomarker has been identified until now.^22^, ^23^, ^24^ We propose that HMGA2 can be used to evaluate the clinical risk of PRCC, though this should be verified in future prospective studies.

Non‐histone chromatin‐associated protein HMGA2 belongs to the HMG protein family. HMG proteins are associated with the functional regulation of DNA and stimulate protein–DNA interactions.^25^ HMGA2, a gene encoding 108 amino acids, is located on chromosome 12q14‐15. Despite having no intrinsic transcriptional activity, this protein acts as an architectural transcription factor that influences gene transcription through chromatin remodeling.^26^, ^27^ As a result of HMGA2, a DNA gap is created at transcription start sites, which is necessary for histone complex formation.^28^ Furthermore, in the absence of DNA, HMGA2 is intrinsically disordered and lacks secondary or tertiary structures.^29^ The DNA‐binding motifs of HMGA2 are traditional unstructured and prefer to bind to AT‐rich DNA fragments and form ordered assemblies. Based on these characteristics, HMGA2 is potentially involved in several biological processes.^30^

HMGA2 is highly expressed in several types of cancer, such as colorectal and renal cell carcinomas, breast cancers, lung cancers, and pancreatic ductal adenocarcinomas,^31^ while a multitude of studies have shown that higher level of expression of HMGA2 is associated with a poorer prognosis and a progression of the disease. A group of target genes is modulated by HMGA2 to promote tumorigenesis, such as SOX‐2, which affects stem‐like cell signaling and induces neuroendocrine prostate cancer progression.^32^ HMGA2 is regulated by miRNAs, among other factors. There has been previous evidence that MiR‐302a‐5p/367‐3p regulates HMGA2, which may serve as a marker for survival for endometrial cancer patients and a therapeutic target for treating it.^33^ In fact, post‐translational modifications of HMGA2 have profound effects on its biological functions, one example is HMGA2 acetylation, which enhances its ability to bind to DNA on target genes while maintaining its stability, it results in HMGA2 accumulation and the progression of esophageal squamous cell carcinoma.^34^ In addition, HMGA2 plays a critical role in immunity, Wang et al. and Wu et al. found that CRC patients with high CD68 or HMGA2 expression had a poorer overall survival rates in CRC patients.^35^, ^36^ Recent research suggests that HMGA2 acts as a downstream target of miR‐103a and promotes proliferation of ccRCC, the most common RCC.^37^ However, the role of HMGA2 in PRCC has not been elucidated. Based on the current study, high expression of HMGA2 appears to be associated with malignancy progression and poor prognosis in PRCC, which is similar to its role as an oncogene in other tumors. However, the underlying mechanism and whether it could be used as a therapeutic target in PRCC remains unclear.

PRCC tumorigenesis is mediated by a variety of molecular mechanisms different from ccRCC tumorigenesis. In a study on the comprehensive molecular characterization of PRCC, alteration of the MET gene (mutation, splice variant, or gene fusion) or gain of chromosome 7 copy number was identified in 81.3% of Type 1 tumor.^38^ Two pathways about PI3K/AKT and MEK/ERK1/2 pathways are important mediators of PRCC tumorigenesis through their roles in promoting cell growth.^39^ Using multiple public genetic datasets, enolase 2 was found to increase glycolysis and cell proliferation, leading to a worse prognosis.^40^ Multi‐omics profiling of PRCC disclosed an increasing glutathione level, which is the main substance to overcome reactive oxygen species. The respiratory chain was significantly downregulated in renal cancer tissue compared with normal renal tissue, following reprogramming of the pathways involved in gluconeogenesis.^41^ Here, through KEGG analysis, we showed that up‐regulated proteins enriched in the spliceosome, ribosome, and RNA transport in PRCC. The main function of the spliceosome is to excise introns and form mature mRNA for translation into proteins, and the ribosome can translate mature mRNA into protein.^42^ RNA transport is essential for protein translation. Since the above three pathways are essential components of gene transcription and translation, we speculate that the change of the proteins in our study may regulate the expression of some key genes related to PRCC above mentioned, thereby promoting the progress of PRCC.

Although our study findings are promising, we acknowledge some limitations. First, we performed immunohistochemical verification for a relatively large number of cases, but the number of samples for proteomic detection was small and may not have included all important biomarkers. Second, we analyzed clinical and pathological parameters of PRCC in relation to immunohistochemical staining for HMGA2, though prospective studies may provide stronger evidence of the clinical outcomes. Third, we only conducted bioinformatics analysis on the possible signaling mechanism of PRCC and need to confirm our prediction in future cytological experiments.

## 5 CONCLUSION

Through proteomics and bioinformatics analyses, we found that HMGA2 was overexpressed in PRCC tumor tissues, and exhibited a shorter survival rate in patients who had high HMGA2 levels. In addition, a close association was found between HMGA2 expression and lymph node metastasis, clinical stage, and tissue subtype. This study demonstrated the HMGA2 to be a potential prognostic biomarker in PRCC for the first time, which could improve patient stratification and inform its clinical management. The function and mechanism of HMGA2 in PRCC require further investigation.

## AUTHOR CONTRIBUTIONS

**Tian Wei:** Data curation (lead); formal analysis (lead); investigation (equal); methodology (lead); writing – original draft (lead). **Huiya Xu:** Investigation (lead); methodology (lead); software (lead); writing – original draft (supporting). **Huishan Liang:** Investigation (supporting); methodology (supporting); resources (supporting). **Yating Lian:** Methodology (supporting); resources (supporting). **Huiyu Chen:** Investigation (supporting); methodology (supporting). **Zhangyi Zheng:** Methodology (supporting); resources (supporting). **Minghui Zhong:** Investigation (supporting); resources (supporting). **Junfeng Liu:** Conceptualization (lead); methodology (supporting); project administration (lead); resources (lead). **Ran Wang:** Validation (equal); writing – review and editing (equal). **Fen Wang:** Conceptualization (lead); data curation (lead); methodology (lead); project administration (lead); resources (lead); supervision (lead); validation (lead); writing – review and editing (lead).

## FUNDING INFORMATION

This research was supported by the National Natural Science Foundation of China (NSFC) (FW, Grant/Award Number: 81972884).

## CONFLICT OF INTEREST STATEMENT

The authors declare they have no conflicts of interest.

## ETHICS STATEMENT

The study was approved by the Institutional Ethics Committee of The First Affiliated Hospital of Sun Yat‐sen University (approval number [2021]404).

## Supporting information


**Figures:** Figure S1A. Figure S1B. Figure S2A. Figure S2B. Figure S2C.

Table S1.

Table S2.

Table S3.

Table S4.

Table S5.

Table S6.

## Data Availability

Data openly available in a public repository.
