# Supplementary material for: Proteomic analysis identifies HMGA2 as a novel biomarker of overall survival in papillary renal cell carcinoma
Source: Cancer Med. 2023 Jun 7;12(13):14851–64. doi: 10.1002/cam4.6077 (PMC10358203; doi:10.1002/cam4.6077)
Supplement: Supplementary file 1 — Figures: Figure S1A. Figure S1B. Figure S2A. Figure S2B. Figure S2C. [file CAM4-12-14851-s005.docx]

**Supplement Figures**


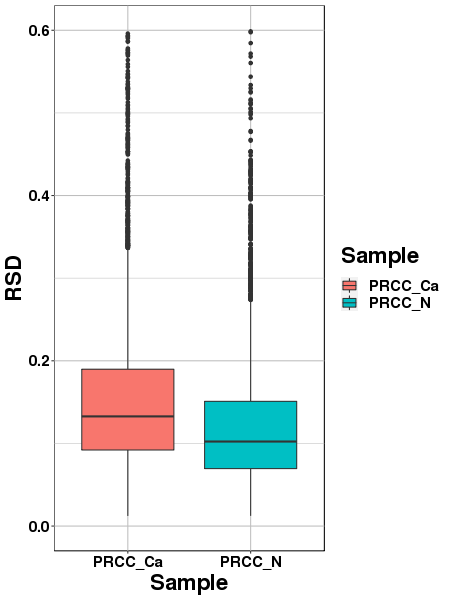


Figure S1A. The box plot of the relative standard deviation (RSD) of protein quantitative values between repeated samples. When the overall RSD value is smaller, the quantitative repeatability is better.


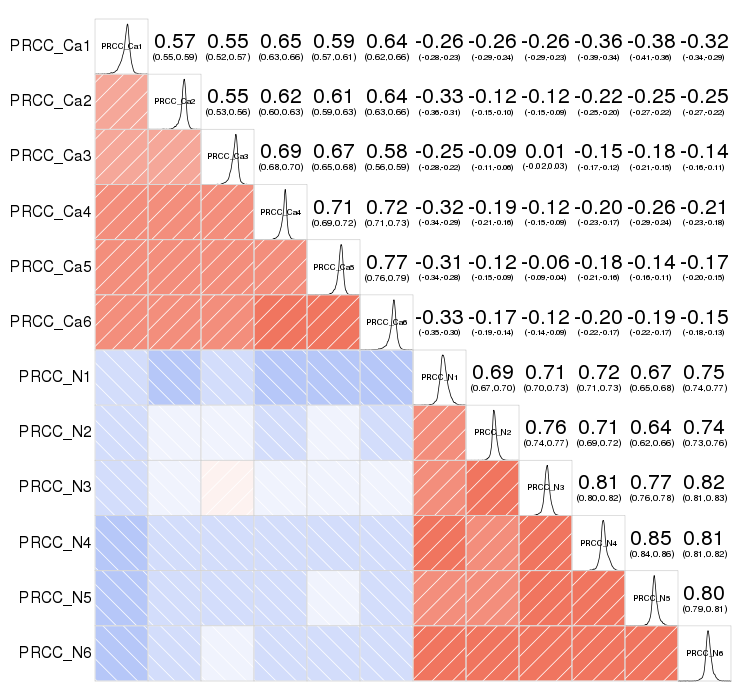


Figure S1B. Pearson’s correlation coefficient for replicate proteome profiling of PRCC-Ca and PRCC-N. This coefficient is a measure of the degree of linear correlation between the two sets of data.


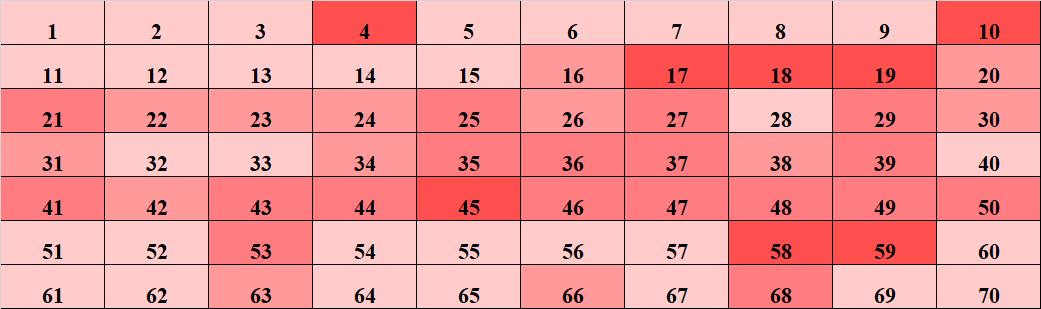


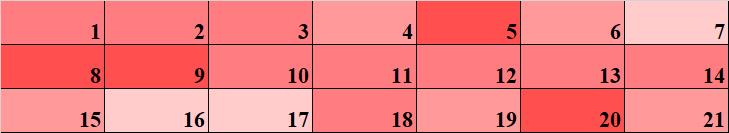


|  |  |  |  |
| --- | --- | --- | --- |
| 0 | 1 | 2 | 3 |

Figure S2A. Immunohistochemical scores of all cases included 70 cases in type 1 PRCC and 21 cases in non-type 1 PRCC. Each square on the way represents a patient, and the darker the color, the higher the score. (All cases were numbered according to sequence of visit).


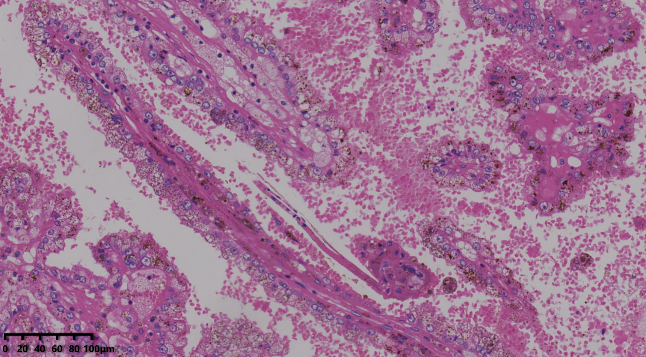

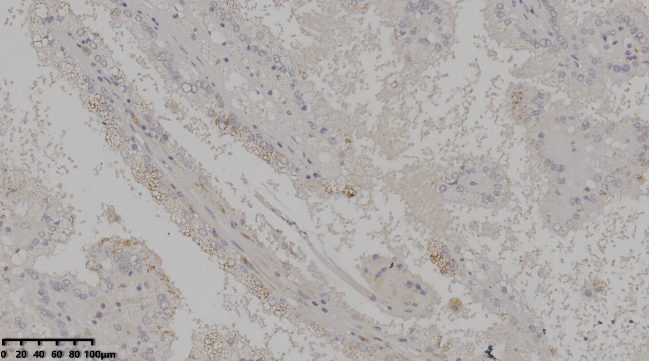


Figure S2B. HE staining showed hemosiderin not really positive, which should not be interpreted as HMGA2 positive. They are granular and expressed in cytoplasm or extracellular. (Scar bar: 100 μm)


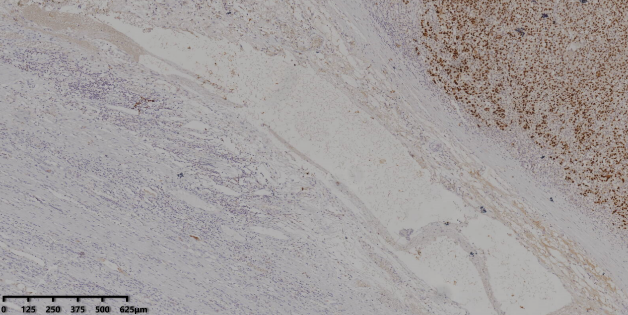

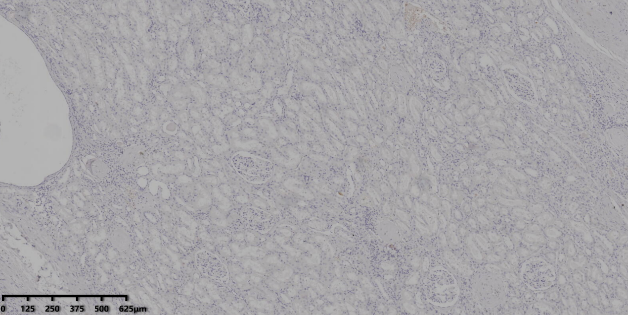


Figure S2C. The negative control was show in normal adjacent tissues. The nuclei of normal tissues were negative. (Scar bar: 625 μm)
